# High origin of a testicular artery: a case report and review of the literature

**DOI:** 10.1186/1752-1947-5-75

**Published:** 2011-02-23

**Authors:** George K Paraskevas, Orestis Ioannidis, Athanasios Raikos, Basileios Papaziogas, Konstantinos Natsis, Ioannis Spyridakis, Panagiotis Kitsoulis

**Affiliations:** 1 Department of Anatomy, Medical School of Aristotle University of Thessaloniki, PO Box 300, Postal Code 54124, Thessaloniki, Greece

## Abstract

**Introduction:**

Although variations in the origin of the testicular artery are not uncommon, few reports about a high origin from the abdominal aorta exist in the literature. We discuss the case of a high origin of the testicular artery, its embryology, classification systems, and its clinical significance.

**Case presentation:**

We report a very rare case of high origin of the left testicular artery in a 68-year-old Caucasian male cadaver. The artery originated from the anterolateral aspect of the abdominal aorta, 2 cm cranially to the ipsilateral renal artery. Approximately 1 cm after its origin, it branched off into the inferior suprarenal artery. During its course, the artery crossed anterior to the left renal artery.

**Conclusions:**

A knowledge of the variant origin of the testicular artery is important during renal and testicular surgery. The origin and course must be carefully identified in order to preserve normal blood circulation and prevent testicular atrophy. A reduction in gonadal blood flow may lead to varicocele under circumstances. A knowledge of this variant anatomy may be of interest to radiologists and helpful in avoiding diagnostic errors.

## Introduction

The testis mainly receives its blood supply from the testicular artery (TA) and drains into the testicular vein [1]. Testicular vessels have an important role in testis thermoregulation [2]. Variations of these arteries and veins have been extensively studied due to their importance in testicular physiology. Moreover, this knowledge has a practical implication during renal and testicular surgery.

Anomalies in the origin, course, and number of TAs were observed in 4.7 percent of cases in a study of 150 cadavers [2]. A high origin of the TA from the abdominal aorta, as in our case report, has been noted in only a few instances in the literature [3-6]. We report on such a case and review the relative literature about the macroscopic anatomy, embryology and likely physiological and surgical implications of this variant.

## Case presentation

**Figure 1:**
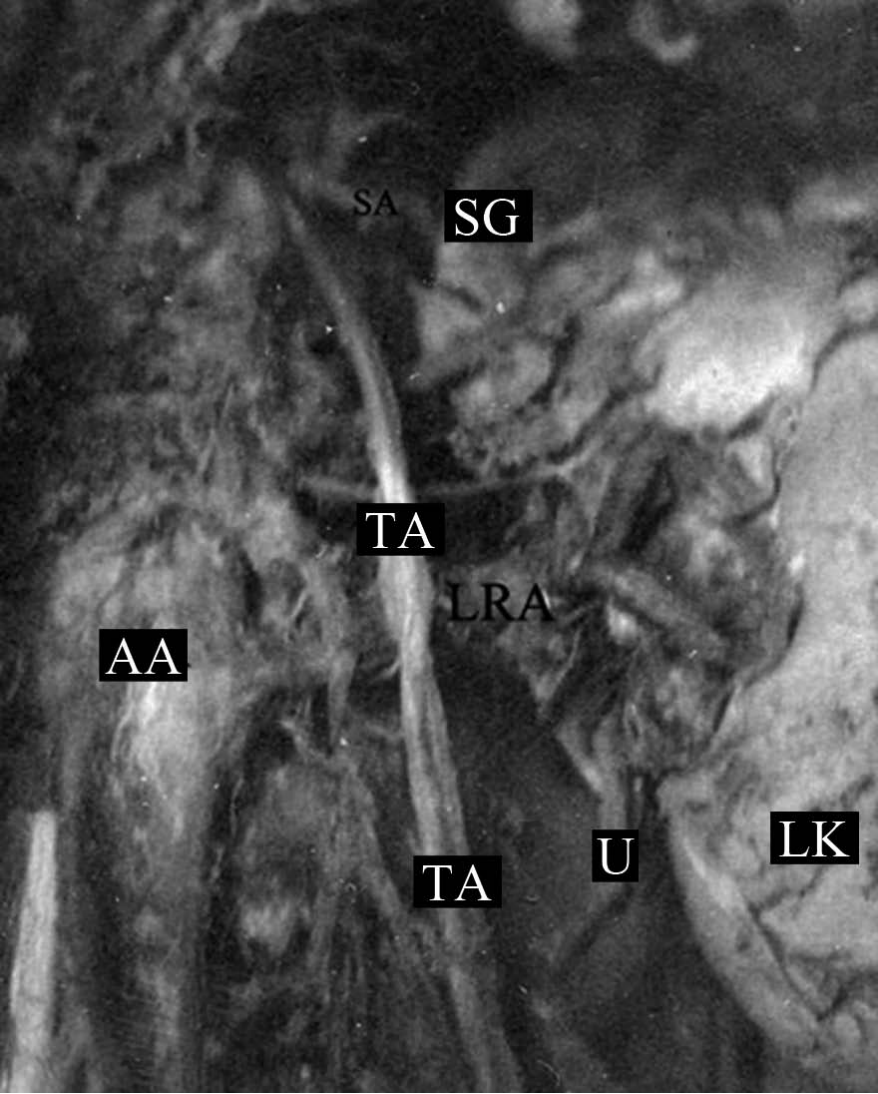
**The left testicular artery (TA) arose from the abdominal aorta (AA), superior to the left renal artery (LRA)**. After its origin, it branched off to the inferior suprarenal artery (SA) and then descended inferiorly, passing over the left renal artery (SG: suprarenal gland, LK: left kidney, U: ureter).

**Figure 2:**
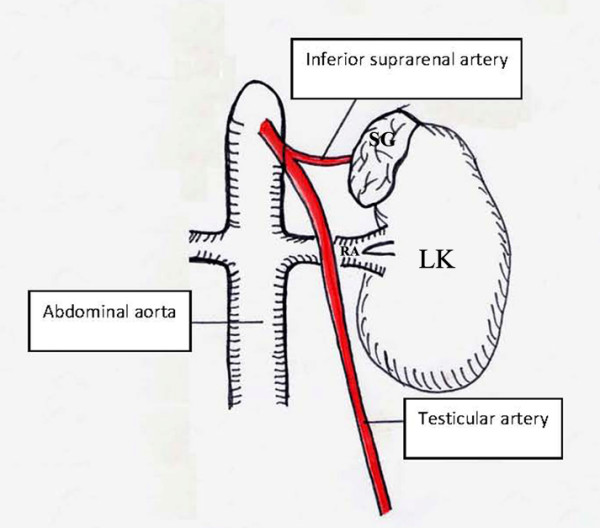
**A schematic representation of Figure 1 (SG: suprarenal gland, LK: left kidney, RA: renal artery)**.

We identified a variation in the origin of the TA in a 68-year-old Caucasian male formalin-embalmed cadaver used for educational and research purposes. His cause of death was cardiovascular ischemic disease. Following dissection of the retro-peritoneum and preparation of the abdominal aorta and its branches, an unusual high origin of the left TA was observed. The artery had a diameter of 32 mm and arose from the anterolateral surface of the abdominal aorta, 2 cm proximal to the ipsilateral renal artery. At 1 cm distal to its origin, it branched off into the inferior suprarenal artery that supplied the left adrenal gland. The left TA then progressed in an oblique course outwards and caudally, crossing anterior to the left renal artery (Figure 1 and Figure 2). His right TA and both the left and right testicular veins were normal.

## Discussion

Anatomical variations of TAs are common. Variants were noticed in 4.7 percent of cases in a study of 150 cadavers [2]. Another study of 90 fetuses revealed a frequency of 8.8 percent [7]. TA variations include variations in the origin, course and even the number of arteries presented. This can include double arteries, a common origin of both arteries, the absence of one artery, a higher origin than normal and origin from the lumbar artery, renal or polar renal, middle or superior suprarenal, common or internal iliac, or superior epigastric artery [2,5,7-11].

TAs are paired and usually originate from the anterolateral or lateral aspect of the abdominal aorta. The TA is a long, thin vessel that arises at an acute angle from the abdominal aorta, at the level of the second lumbar vertebra below the renal artery [1]. Each TA passes inferolaterally under the parietal peritoneum and over the psoas major muscle. The right TA lies anterior to the inferior vena cava and posterior to the third portion of the duodenum, while the left lies posterior to the lower part of the descending colon [12]. In rare instances, the right TA passes posterior to the inferior vena cava [13].

In both men and women, the abdominal portion of the TA (ovarian in females) seems to have the same topographical relationship. Along its course, the TA supplies anatomical structures such as the peritoneum and profound inguinal ring, perirenal fat, ureter, iliac lymph, retroperitoneum, spermatic cord and cremaster muscle. Sometimes the TA branches off to the inferior pole of the ipsilateral adrenal gland [1,12,13].

There are few reports of a high TA origin in the literature. Shinohara *et al. *found a TA originating 1 cm superior to the origin of the inferior phrenic artery [3]. After a short course, it branched off and subdivided into a supernumerary inferior phrenic artery and a superior suprarenal artery. In another case, Onderoglu *et al. *reported the case of a high origin of the right TA located at the level of the right renal artery lineage [4]. It branched off and was subdivided into an inferior phrenic artery and a superior suprarenal artery. In another study, Brohi *et al. *described the case of a high origin of the left TA which originated from the left renal artery [5]. The artery branched off and was subsequently subdivided into three branches that supplied the left suprarenal gland. Two more cases of a higher origin of the TA were reported by Ozan *et al. *[6]. Furthermore, Xue *et al. *found a right TA artery arising from the anterior surface of the abdominal aorta at the level of the left renal artery [14].

The first attempt at classification of TA variations was made by Machnicki *et al. *[15]. Their study included TAs from both fetuses and adults grouped according to their origin from the aorta or renal artery. Four major types were observed: Type A - a single TA originating from the aorta; Type B - a single TA originating from the renal artery; Type C - two TAs originating from the aorta that supplied the same gonad; Type D - two TAs supplying the same gonad, one arising from the aorta and the other from the renal artery [15]. Some years later, Çiçekcibasi *et al. *classified the variations into four alternative types: Type I - TA arising from the suprarenal artery; Type II - TA originating from the renal artery; Type III - TA of high-positional origin from the abdominal aorta, close to the renal artery lineage; Type IV - TA duplication originating from the aorta or from various vessels [7]. Our case report is Type A, according to classification by Machnicki *et al. *[15] and Type III, according to classification by Çiçekcibasi *et al. *[7].

Notkovich described the relationship of the TA to the renal vein [16]. In his study, the anatomical variations are divided into three types: Type I - TA arising from the aorta, passing posterior or inferior to the renal vein but without making contact with it; Type II - TA originating from the aorta, superior to the renal vein and crossing in front of it; Type III - TA arising from the aorta and passing posterior or inferior to the renal vein and coursing superiorly and around the renal vein [16]. Our case report is classified as Type II according to Notkovich classification.

The ratio of common origin for the TA and the suprarenal artery is approximately 1:26 [17]. The superior suprarenal artery usually arises from the inferior phrenic artery, the middle suprarenal artery arises from the abdominal aorta and the inferior suprarenal artery from the renal artery [1,10]. Although anatomical variations of the middle suprarenal artery are common [18], reports of variations of the inferior and superior suprarenal arteries are rare [2,19]. The phenomenon of a common origin for both the testicular and suprarenal artery has also been described [20,21].

Variations in the origin, course and branches of TAs are attributed to their embryologic derivation. Felix proposed that there are nine lateral mesonephric arteries in an 18 mm embryo and that they are grouped as follows: 1) the cranial group, which is made up of the first and second mesonephric arteries that are located proximal to the celiac trunk of the abdominal aorta and directed posterior to the suprarenal gland; 2) the middle group, which is made up of the third to fifth mesonephric arteries which run along the ventral surface of the suprarenal gland; 3) the caudal group, which is made up of the sixth to ninth mesonephric arteries which run along to the ventral surface of the suprarenal gland [22]. The caudal group forms the arterial plexus of the urogenital system [22,23].

Despite the fact that any of the nine mesonephric arteries can evolve to become the TA, Felix reported that the TA usually derives from the caudal group. In the same study, Felix claimed that the TA rarely derives from the cranial group. When such a case occurs, the TA is brought posteriorly to the renal artery, which originates from the middle group. In our case report, the TA corresponds to the cranial group as it is located superior to the celiac trunk [22]. However, in our case report, and contrary to Felix's report, the TA is located anterior to the renal artery. This means that the cranial and caudal groups are not necessarily independent of each other but connected by longitudinal anastomotic channels located ventrally to the developing renal artery.

During developmental modifications of the gastrointestinal tract, the celiac splanchnic arteries and their longitudinal anastomotic channels are gradually disappearing. This leads to anatomical variations of the celiac, superior and inferior mesenteric arteries. Likewise, various disappearing phases of the lateral mesonephric arteries and their longitudinal anastomotic channels can take place during the embryonic development of the gonads. These modifications can lead to variants of the suprarenal, renal and testicular arteries. The persistence of many mesonephric arteries may lead to multiple testicular arteries [24].

The anatomical variations of TAs are of clinical importance as well as embryological and anatomical interest. Practical implications can be found in the kidney and gonad blood flow. Such conditions could lead to varicocele under circumstances [16]. The variant becomes more significant in light of the fact that testicular arterial blood flow was found to be significantly decreased in men with varicocele [25]. Additionally, anomalous TA origin may affect the testicular perfusion and testicular function. Since age-related disturbances in spermiogenesis are well described in the literature, it would be wise for the clinician to differentially diagnose age-related impaired spermiogenesis from perfusion-induced spermiogenesis.

## Conclusions

Anatomical knowledge of the origin and course of the TA is of great importance during renal and testicular surgery. The origin and course of the TA must be carefully identified and demarcated in order to preserve and prevent testicular atrophy. Aside from surgical interest, the trait is of clinical value because anomalies in arterial and venous perfusion may have severe consequences for the thermoregulation of the testicular glands and may therefore influence spermiogenesis. Furthermore, radiologists should be familiar with TA variants in order to provide an accurate diagnosis during pre-clinical studies.

## Abbreviations

TA: Testicular artery.

## Consent

Written informed consent was obtained from the patient's next-of-kin for publication of this case report and any accompanying images. A copy of the written consent is available for review by the Editor-in-Chief of this journal.

## Competing interests

The authors declare that they have no competing interests.

## Authors' contributions

GKP identified the variant, performed the anatomical dissection, created the schematic drawing and reviewed the final version of the manuscript. OI and AR prepared the draft of the manuscript. AR improved the image presented in this report. BP, KN, IS, and PK performed the final edit of the manuscript. All authors read and approved the final manuscript.
